# Supplementary material for: In Silico Multi-Target Approach Revealed Potential Lead Compounds as Scaffold for the Synthesis of Chemical Analogues Targeting SARS-CoV-2
Source: Biology (Basel). 2022 Mar 18;11(3):465. doi: 10.3390/biology11030465 (PMC8945274; doi:10.3390/biology11030465)
Supplement: Supplementary file 1 [file biology-11-00465-s001.zip › biology-1613033-supplementary.pdf]

Supplementary Materials

Synthesis and analytical data for COR267, COR1393 and COR1461

COR267 was prepared starting from 1 [1] (Scheme 1) by alkylation with 2-(bromomethyl) naphthalene in the presence of tetrabutylammonium bromide as phase-transfer catalyst.

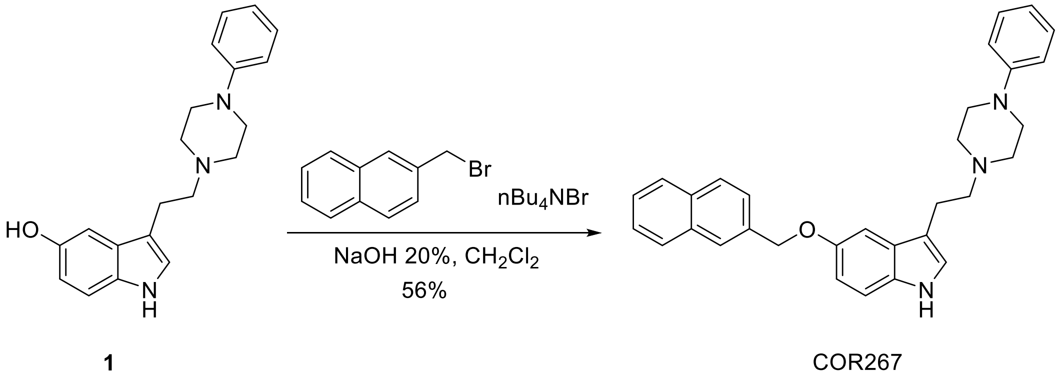

Scheme S1. Synthesis of COR267

COR1393 was synthesized starting from **2** [2] (Scheme 2) by amidation reaction in the presence of picolinic acid.

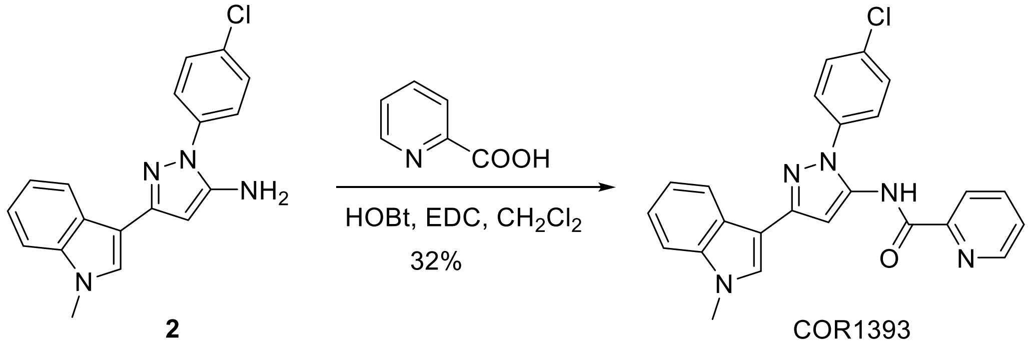

Scheme S2. Synthesis of COR1393

COR1461 was obtained by a multistep reaction reacting **3** [2] (Scheme 3) with 3-bromobenzaldehyde and 5,5-dimethylcyclohexan-1,3-dione in acetic acid.

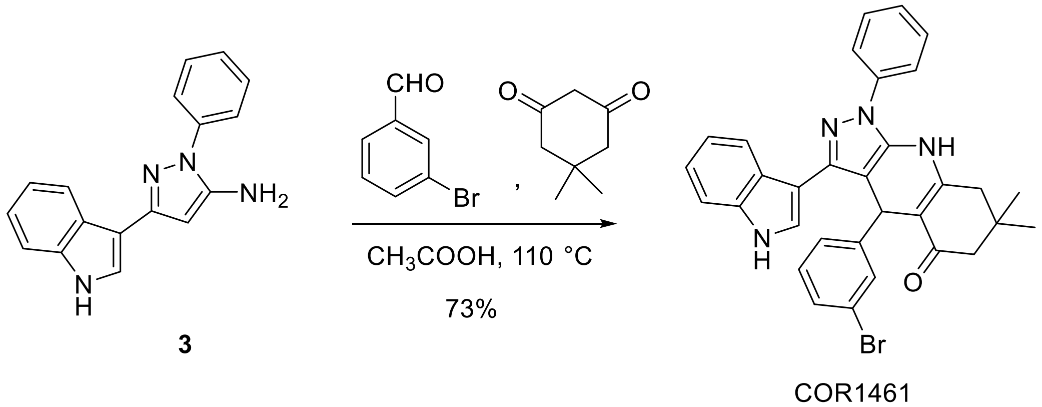

Scheme S3. Synthesis of COR1461

5-(Naphthalen-2-ylmethoxy)-3-(2-(4-phenylpiperazin-1-yl)ethyl)-1*H*-indole (COR267).

To a solution of **1** (60 mg, 0.19 mmol) in CH_2Cl_2 (3 mL) 20% NaOH (2 mL), *n*BuN^+Br^- (30 mg, 0.09 mmol) and 2-(bromomethyl)naphthalene (45 mg, 0.2 mmol) were added. The reaction mixture was stirred vigorously for 30 min, then extracted with CH_2Cl_2. The organic phase was dried over anhydrous Na_2SO_4, the solvent removed under reduced pressure and the residue purified by flash chromatography (eluent: CH_2Cl_2/MeOH 98:2) to give COR 267 (48 mg, 0.10 mmol) as a white solid. Mp 126-128 °C. ^{1}H NMR (400 MHz, $CDCl_3$): δ 7.94 (s, 1H), 7.92-7.82 (m, 3H), 7.61 (d, *J* = 8.4 Hz, 1H), 7.51-7.46 (m, 2H), 7.31-7.25 (m, 3H), 7.19 (d, *J* = 2.1 Hz, 1H), 7.03 (s, 1H), 6.99 (dd, *J* = 8.8 Hz, *J* = 2.3 Hz, 1H), 6.96 (d, *J* = 8.1 Hz, 2H), 6.88 (t, *J* = 7.3 Hz, 1H), 5.30 (s, 2H), 3.30-3.20 (m, 4 H), 2.98-2.94 (m, 2H), 2.73-2.68 (m, 6H). ^{13}C NMR (100 MHz, $CDCl_3$): δ 153.1, 151.3, 132.2, 133.3, 133.0, 131.6, 129.1, 128.3, 127.9, 127.8, 127.7, 126.2, 126.1, 125.9, 125.4, 122.4, 119.7, 116.0, 114.1, 112.9, 111.8, 102.7, 71.2, 59.1, 53.2, 49.1, 22.9. MS (ESI): *m*/*z* 462 $[M+H]^+$ (100).

***N*-(1-(4-Chlorophenyl)-3-(1-methyl-1*H*-indol-3-yl)-1*H*-pyrazol-5-yl)picolinamide** (COR1393). To a solution of **2** (100 mg, 0.3 mmol) in CH_2Cl_2 (10 mL) EDC (102 mg, 0.5 mmol), HOBt (72 mg, 0.5 mmol) and picolinic acid (58 mg, 0.5 mmol) were added. The reaction mixture was stirred at room temperature for 18 h, then it was washed with aqueous $NaHCO_3$, dried over anhydrous Na_2SO_4 and the solvent evaporated under reduced pressure. The brown oil thus obtained was purified by flash chromatography (eluent PE/EtOAc 2:1) to give a solid which was recrystallized from EtOAc. COR1393 (41 mg, 0.1 mmol) was obtained as a yellow solid. Mp 201–204 °C. ^{1}H NMR (400 MHz, $CDCl_3$): δ 10.41 (s, 1H), 8.50 (d, *J* = 4.5 Hz, 1H), 8.21 (dd, *J* = 22.4, 7.8 Hz, 2H), 7.88 (t, *J* = 7.7 Hz, 1H), 7.68 (s, 1H), 7.62 (d, *J* = 8.5 Hz, 2H), 7.52 (s, 1H), 7.45 (dd, *J* = 7.5, 4.9 Hz, 1H), 7.36–7.07 (m, 3H), 3.80 (s, 3H). ^{13}C NMR (100 MHz, $CDCl_3$): δ 160.7, 148.8, 148.6, 148.4, 137.9, 137.3, 137.1, 136.1, 133.4, 130.0, 127.5, 127.0, 126.0, 125.4, 122.6, 122.1, 121.6, 120.2, 109.3, 108.7, 95.4, 33.0. MS (ESI): *m*/*z* 428 $[M+H]^+$ (100).

4-(3-Bromophenyl)-3-(1*H*-indol-3-yl)-7,7-dimethyl-1-phenyl-1,4,6,7,8,9-hexahydro-5*H*-pyrazolo[3,4-b]quinolin-5-one (COR1461)**.** To a solution of **3** (200 mg, 0.7 mmol) in glacial acetic acid kept at 70 °C, 5,5-dimethylcyclohexan-1,3-dione (102 mg, 0.7 mmol) and 3-bromobenzaldehyde (129 mg, 0.7 mmol) were added and the reaction mixture was refluxed for 2 h. After cooling, the reaction mixture was poured into water and the yellow precipitate was extracted with AcOEt. The organic phase was washed with 10% Na_2CO_3 and dried over anhydrous Na_2SO_4. After removal of the solvent, the yellow oil was purified by flash chromatography (eluent: CH_2Cl_2) to give COR1461 (287 mg, 0.51 mmol) as a yellow solid. Mp > 270 °C. ^{1}H (400 MHz, $CDCl_3$): δ 8.40 (d, *J* = 7.6 Hz, 1H), 7.93 (s, 1H), 7.86 (d, *J* = 7.8 Hz, 1H), 7.51 (t, *J* = 8.0 Hz, 4H), 7.24 (ddd, *J* = 21.5, 8.7, 4.3 Hz, 4H), 7.11 (dd, *J* = 20.5, 4.2 Hz, 3H), 6.94 (dd, *J* = 8.9, 4.4 Hz, 3H), 6.04 (d, *J* = 2.6 Hz, 1H), 3.23 (s, 2H), 2.50 (s, 2H), 1.11 (d, *J* = 2.7 Hz, 6H). ^{13}C NMR (100 MHz, DMSO–*d6*): δ 197.1, 163.7, 150.3, 148.8, 143.4, 139.9, 139.8, 136.1, 131.3, 130.8, 129.9, 129.7, 127.9, 126.7, 126.6, 122.1, 121.4, 121.0, 120.7, 120.1, 114.8, 111.8, 106.8, 53.9, 48.2, 48.1, 32.5, 28.2. MS (ESI): *m*/*z* 562 $[M-H]^-$ (100).

**Compound**	**2D Structure**
**COR267**	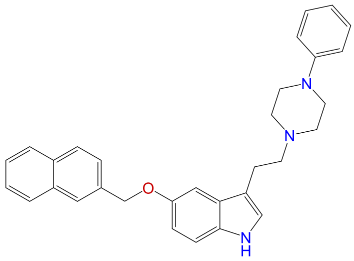
**COR437**	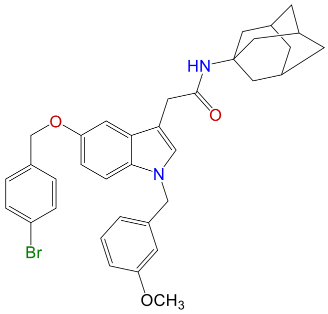
**COR480**	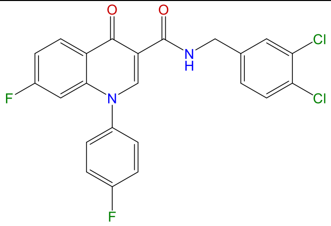
**COR482**	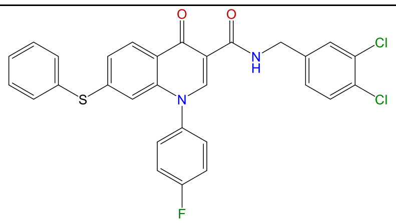
**COR483**	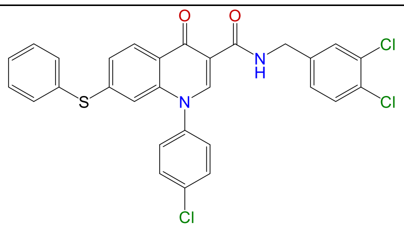
**COR 1393**	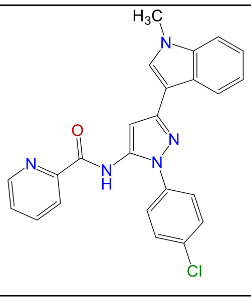

**COR1461**	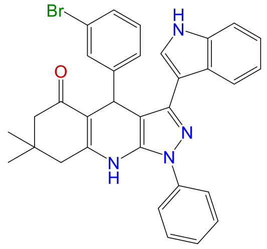

Table S1. The 2D structures of best compounds selected by the virtual screening process.

Ranking	Compound	Binding free energy (Kcal/mol)
1	COR 480	-8.8
2	COR 482	-8.8
3	COR 483	-8.3
4	COR 925	-8.3
5	COR 485	-8.2
6	COR 484	-8.1
7	COR 248	-8.0
8	COR 264	-8.0
9	COR 570	-8.0
10	COR 572	-7.9
11	COR 1463	-7.8
12	COR 1507	-7.8
13	COR 270	-7.8
14	COR 332	-7.8
15	COR 472	-7.8
16	COR 571	-7.8
17	COR 632	-7.8
18	COR 1473	-7.7
19	COR 219	-7.7
20	COR 239	-7.7
21	COR 244	-7.7
22	COR 440	-7.7
23	COR 442	-7.7
24	COR 476	-7.7
25	COR 606	-7.7
26	COR 1509	-7.6
27	COR 1539	-7.6
28	COR 249	-7.6
29	COR 268	-7.6
30	COR 436	-7.6
31	COR 510	-7.6
32	COR 516	-7.6
33	COR 620	-7.6
34	COR 633	-7.6
35	COR 815	-7.6

36	COR 918	-7.6
37	COR 120	-7.5
38	COR 1587	-7.5
39	COR 215	-7.5
40	COR 262	-7.5
41	COR 274	-7.5
42	COR 275	-7.5
43	COR 321	-7.5
44	COR 346	-7.5
45	COR 348	-7.5
46	COR 377	-7.5
47	COR 435	-7.5
48	COR 438	-7.5
49	COR 488	-7.5
50	COR 563	-7.5
51	COR 607	-7.5
52	COR 614	-7.5
53	COR 809	-7.5
54	COR 1218	-7.4
55	COR 1219	-7.4
56	COR 1516	-7.4
57	COR 178	-7.4
58	COR 226	-7.4
59	COR 227	-7.4
60	COR 228	-7.4
61	COR 235	-7.4
62	COR 241	-7.4
63	COR 243	-7.4
64	COR 267	-7.4
65	COR 441	-7.4
66	COR 564	-7.4
67	COR 573	-7.4
68	COR 577	-7.4
69	COR 597	-7.4
70	COR 775	-7.4
71	COR 915	-7.4
72	COR 1151	-7.3
73	COR 1220	-7.3
74	COR 1222	-7.3
75	COR 1319	-7.3
76	COR 1469	-7.3
77	COR 1498	-7.3
78	COR 1556	-7.3
79	COR 1616	-7.3
80	COR 1617	-7.3
81	COR 205	-7.3
82	COR 220	-7.3

83	COR 236	-7.3
84	COR 240	-7.3
85	COR 273	-7.3
86	COR 318	-7.3
87	COR 335	-7.3
88	COR 390	-7.3
89	COR 418	-7.3
90	COR 530	-7.3
91	COR 555	-7.3
92	COR 567	-7.3
93	COR 612	-7.3
94	COR 616	-7.3
95	COR 731	-7.3
96	COR 733	-7.3
97	COR 808	-7.3
98	COR 869	-7.3
99	COR 938	-7.3
100	COR 990	-7.3
101	COR 1288	-7.2
102	COR 1466	-7.2
103	COR 1472	-7.2

Table S2. Distribution of top 103 binding free energy scores on the SARS-CoV-2 S-glycoprotein trimerization region

Ranking	Compound	Binding free energy (Kcal/mol)
1	COR 480	-8.7
2	COR 482	-8.6
3	COR 1393	-8.5
4	COR 1472	-8.5
5	COR 490	-8.3
6	COR 609	-8.3
7	COR 1200	-8.2
8	COR 488	-8.2
9	COR 639	-8.2
10	COR 925	-8.2
11	COR 1480	-8.1
12	COR 476	-8.1
13	COR 1518	-8.0
14	COR 1528	-8.0
15	COR 262	-8.0
16	COR 430	-8.0
17	COR 475	-8.0
18	COR 571	-8.0
19	COR 918	-8.0
20	COR 1522	-7.9
21	COR 1540	-7.9
22	COR 1542	-7.9

23	COR 1587	-7.9
24	COR 267	-7.9
25	COR 274	-7.9
26	COR 359	-7.9
27	COR 445	-7.9
28	COR 471	-7.9
29	COR 485	-7.9
30	COR 487	-7.9
31	COR 849	-7.9
32	COR 883	-7.9
33	COR 1163	-7.8
34	COR 1218	-7.8
35	COR 1509	-7.8
36	COR 1520	-7.8
37	COR 1568	-7.8
38	COR 1617	-7.8
39	COR 226	-7.8
40	COR 235	-7.8
41	COR 239	-7.8
42	COR 377	-7.8
43	COR 392	-7.8
44	COR 404	-7.8
45	COR 439	-7.8
46	COR 472	-7.8
47	COR 489	-7.8
48	COR 491	-7.8
49	COR 554	-7.8
50	COR 570	-7.8
51	COR 607	-7.8
52	COR 614	-7.8
53	COR 632	-7.8
54	COR 633	-7.8
55	COR 879	-7.8
56	COR 915	-7.8
57	COR 1211	-7.7
58	COR 1215	-7.7
59	COR 1319	-7.7
60	COR 1535	-7.7
61	COR 237	-7.7
62	COR 240	-7.7
63	COR 289	-7.7
64	COR 332	-7.7
65	COR 394	-7.7
66	COR 539	-7.7
67	COR 556	-7.7
68	COR 572	-7.7
69	COR 606	-7.7

70	COR 616	-7.7
71	COR 660	-7.7
72	COR 731	-7.7
73	COR 830	-7.7
74	COR 92	-7.7
75	COR 1219	7.6
76	COR 1222	7.6
77	COR 1253	7.6
78	COR 1269	7.6
79	COR 1516	7.6
80	COR 1541	7.6
81	COR 1553	7.6
82	COR 1555	7.6
83	COR 1641	7.6
84	COR 167	7.6
85	COR 178	7.6
86	COR 238	7.6
87	COR 241	7.6
88	COR 265	7.6
89	COR 275	7.6
90	COR 437	7.6
91	COR 498	7.6
92	COR 514	7.6
93	COR 59	7.6
94	COR 615	7.6
95	COR 707	7.6
96	COR 847	7.6
97	COR 875	7.6
98	COR 91	7.6
99	COR 1039	-7.5
100	COR 1048	-7.5
101	COR 1162	-7.5
102	COR 120	-7.5
103	COR 121	-7.5

Table S3. Distribution of top 103 binding free energy scores on the SARS-CoV-2 RBD/hACE2 interaction region

**Ranking**	**Compound**	**Binding free energy (Kcal/mol)**
1	COR 267	-9.4
2	COR 437	-8.8
3	COR 1461	-8.8
4	COR 270	-8.8
5	COR 441	-8.8
6	COR 478	-8.8
7	COR 1542	-8.7
8	COR 476	-8.7
9	COR 607	-8.7

10	COR 1039	-8.6
11	COR 472	-8.6
12	COR 475	-8.6
13	COR 484	-8.6
14	COR 606	-8.6
15	COR 1473	-8.5
16	COR 440	-8.5
17	COR 485	-8.5
18	COR 571	-8.5
19	COR 577	-8.5
20	COR 730	-8.5
21	COR 1641	-8.4
22	COR 393	-8.4
23	COR 487	-8.4
24	COR 509	-8.4
25	COR 1319	-8.3
26	COR 1525	-8.3
27	COR 167	-8.3
28	COR 228	-8.3
29	COR 366	-8.3
30	COR 392	-8.3
31	COR 444	-8.3
32	COR 471	-8.3
33	COR 474	-8.3
34	COR 477	-8.3
35	COR 486	-8.3
36	COR 505	-8.3
37	COR 564	-8.3
38	COR 1466	-8.2
39	COR 1518	-8.2
40	COR 1541	-8.2
41	COR 1568	-8.2
42	COR 183	-8.2
43	COR 227	-8.2
44	COR 368	-8.2
45	COR 442	-8.2
46	COR 443	-8.2
47	COR 447	-8.2
48	COR 488	-8.2
49	COR 490	-8.2
50	COR 514	-8.2
51	COR 588	-8.2
52	COR 595	-8.2
53	COR 731	-8.2
54	COR 1483	-8.1
55	COR 1556	-8.1
56	COR 215	-8.1

57	COR 219	-8.1
58	COR 271	-8.1
59	COR 273	-8.1
60	COR 274	-8.1
61	COR 450	-8.1
62	COR 491	-8.1
63	COR 532	-8.1
64	COR 593	-8.1
65	COR 610	-8.1
66	COR 616	-8.1
67	COR 1040	-8.0
68	COR 1113	-8.0
69	COR 1313	-8.0
70	COR 142	-8.0
71	COR 1509	-8.0
72	COR 1539	-8.0
73	COR 155	-8.0
74	COR 205	-8.0
75	COR 248	-8.0
76	COR 342	-8.0
77	COR 367	-8.0
78	COR 424	-8.0
79	COR 437	-8.0
80	COR 445	-8.0
81	COR 473	-8.0
82	COR 480	-8.0
83	COR 497	-8.0
84	COR 498	-8.0
85	COR 508	-8.0
86	COR 535	-8.0
87	COR 562	-8.0
88	COR 574	-8.0
89	COR 576	-8.0
90	COR 605	-8.0
91	COR 1041	-7.9
92	COR 1068	-7.9
93	COR 1480	-7.9
94	COR 161	-7.9
95	COR 1675	-7.9
96	COR 16	-7.9
97	COR 184	-7.9
98	COR 226	-7.9
99	COR 240	-7.9
100	COR 247	-7.9
101	COR 332	-7.9
102	COR 377	-7.9
103	COR 423	-7.9

Table S4. Distribution of top 103 binding free energy scores on the SARS-CoV-2 3CLpro.

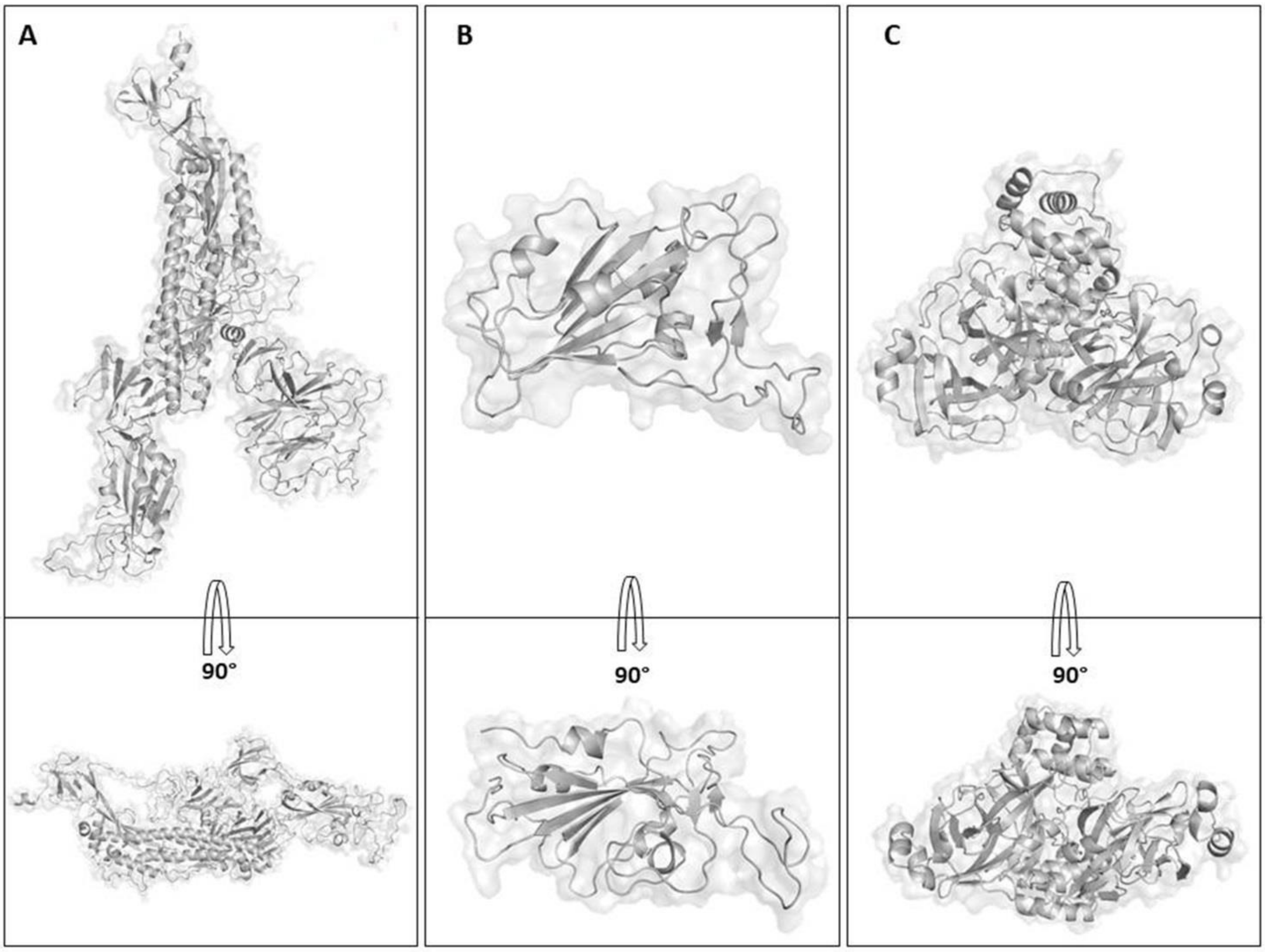

Figure S1. Snapshot of SARS-CoV-2 S-glycoprotein trimer, S-Protein RBD domain and 3CLpro 3D models used for docking and MD simulations. Each biological system is represented as a transparent grey surface cartoon. In panels A, B and C are reported the SARS-CoV-2 S-glycoprotein trimer, S-Protein RBD domain and 3CLpro 3D structures of SARS-CoV-2, respectively. The 3D structures shown below in each panel represent the same structure rotated of 90° around the y-axis on the original construction plane.

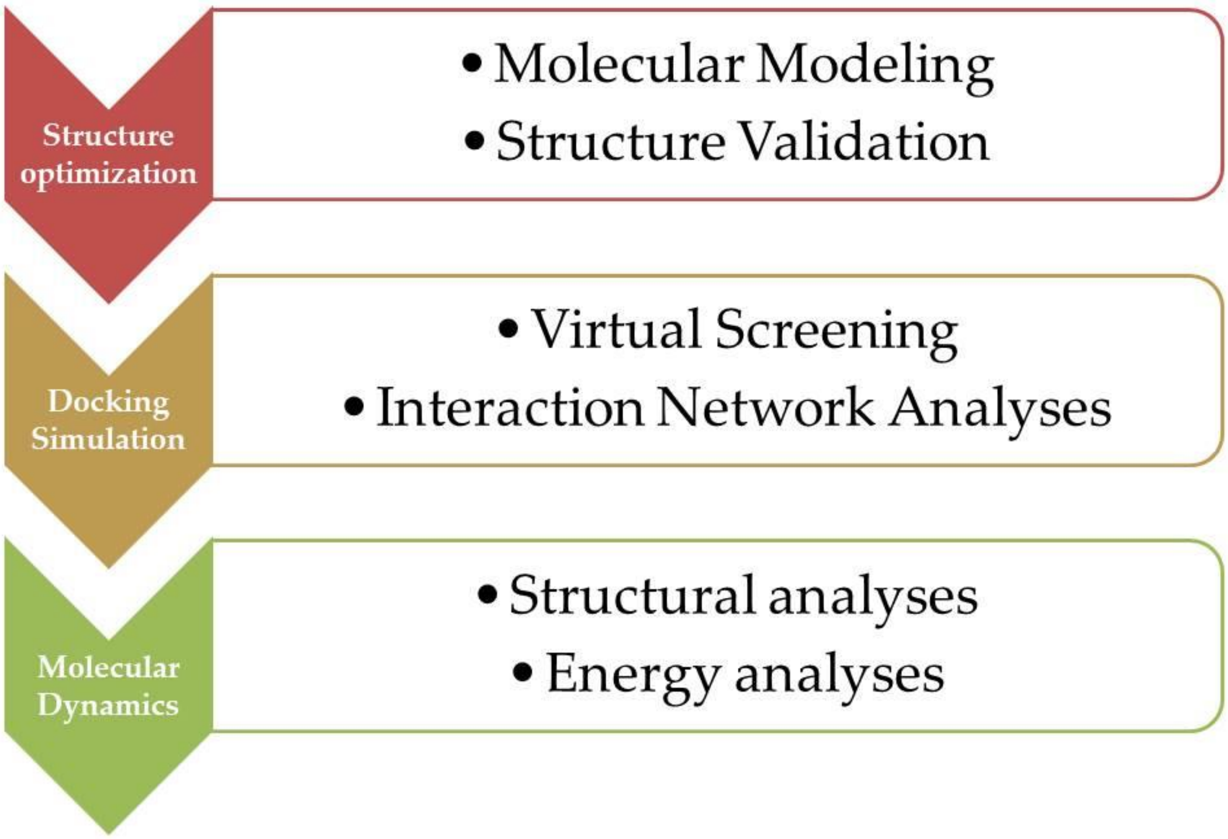

Figure S2. Computational Workflow.

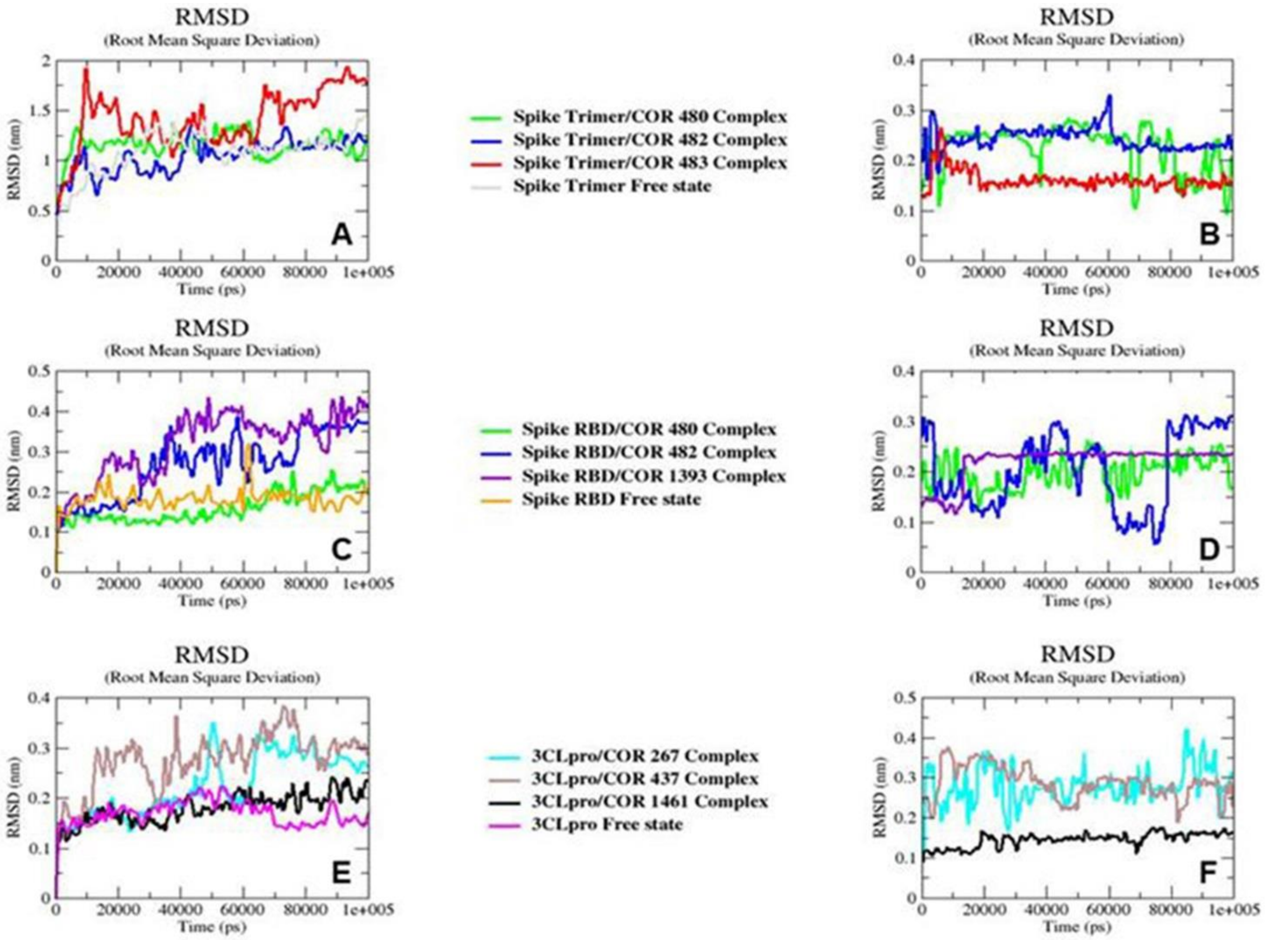

Figure S3. Root mean square deviation (RMSD) plots. **(A)** The RMSD profile of Spike Trimer backbone and **(B)** COR 480, COR 482 and COR 483 ligands. **(C)** The RMSD trend of Spike RBD backbone and **(D)** COR 480, COR 482 and COR 1393 compounds. **(E)** The RMSD of 3CLpro backbone and **(F)** COR 267, COR 437 and COR 1461 chemicals. Each RMSD analyse was relative to the initial frame against simulation time.

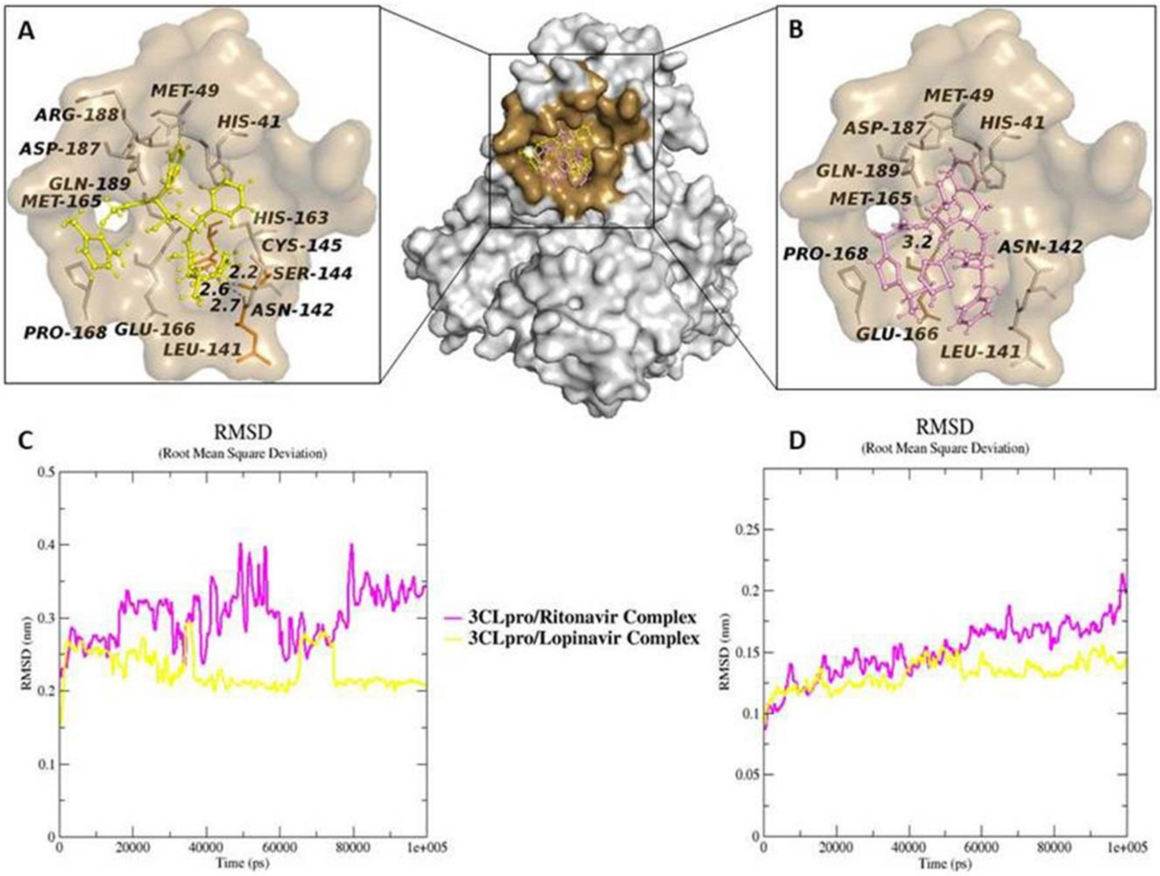

Figure S4. Surface representation of the 3CLpro structure in complex with Lopinavir and Ritonavir and Root Mean Square Deviation (RMSD) plots. (A-B) The pocket surface patch is depicted in brown, while Lopinavir and Ritonavir are shown in yellow and pink balls and sticks, respectively. Residues forming direct interactions with the ligands are shown as grey (Hydrophobic interaction) and orange (Hydrogen bond) balls and sticks. Hydrogen bonds are indicated with pink dashed lines. The number closely the dashed lines, it represents the bond length. (C) RMSD trend of 3CLpro backbone. (D) RMSD profile of Lopinavir and Ritonavir. Each RMSD analyse was relative to the initial frame against simulation time.

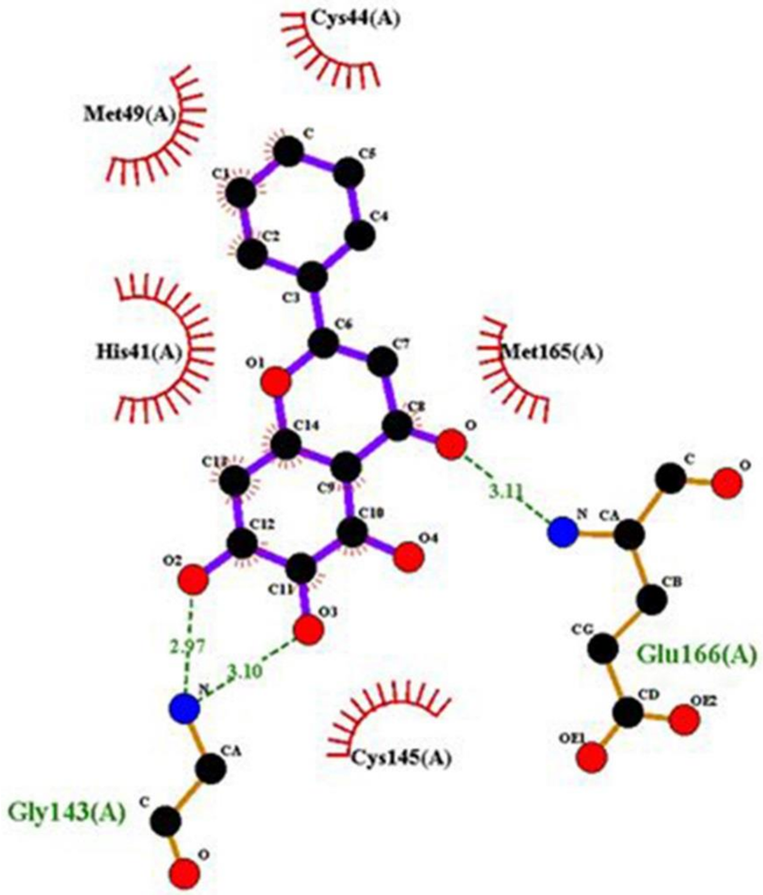

Figure S5. 3CLpro-Baicalein interaction network. Baicalein is shown in purple balls and sticks, while the residues involved in hydrophobic interactions and hydrogen bonds are represented in red lines and brown balls and sticks. The hydrogen bonds are indicated as green dashed lines. The number over the dashed lines, it represents the hydrogen bond length.

Compound	Target	In silico method	Docking score (Kcal/mol)	Reference
**COR 480**	**STR***	**Docking**	**-8.8**	p.w.*
**COR 482**	**STR***	**Docking**	**-8.8**	p.w.*
**COR 483**	**STR***	**Docking**	**-8.3**	p.w.*
Ergoloid	STR*	Docking	−8.0	18
Darifenacin	STR*	Docking	−7.7	18
5-methyltetrahydrofolic acid	STR*	Docking	−7.7	18
Buclizine	STR*	Docking	−7.6	18
Saquinavir	STR*	Docking	−7.5	18
Solifenacin	STR*	Docking	−7.5	18
Sorafenib	STR*	Docking	−7.4	18
Tetrahydrofolic acid	STR*	Docking	−7.4	18
**COR 480**	**S-RBD***	**Docking**	**-8.7**	p.w.*
**COR 482**	**S-RBD***	**Docking**	**-8.6**	p.w.*
**COR 1393**	**S-RBD***	**Docking**	**-8.5**	p.w.*
Apigenin	S-RBD*	Docking	-7.8	42
Chrysin	S-RBD*	Docking	-8.1	42
Fisetin	S-RBD*	Docking	-8.3	42
Galangin	S-RBD*	Docking	-8.2	42
Hesperetin	S-RBD*	Docking	-7.7	42
Luteolin	S-RBD*	Docking	-8	42
Morin	S-RBD*	Docking	-8.1	42
Quercetin	S-RBD*	Docking	-8.2	42
Dexamethasone	S-RBD*	Docking	-7.9	42
Nüzhenide oleoside	S-RBD*	Docking	-8.9	45
Oleuropein dimer	S-RBD*	Docking	-8.7	45
Dihydro oleeuropein	S-RBD	Docking	-8.7	45
Chloroquine	S-RBD	Docking	-5.7	45
**COR 267**	**3CLpro**	**Docking**	**-9.4**	p.w.*
**COR 437**	**3CLpro**	**Docking**	**-8.8**	p.w.*
**COR 1461**	**3CLpro**	**Docking**	**-8.8**	p.w.*
Altertoxin V	3CLpro	Docking	−7.2	44
Altertoxin II	3CLpro	Docking	−7.5	44
Penicillixanthone A	3CLpro	Docking	−8.2	44
Cytochalasin Z8	3CLpro	Docking	−7.9	44
Stachybotrosin D	3CLpro	Docking	−6.5	44
Chloropupukeanolide A	3CLpro	Docking	−7.5	44
Phomasetin	3CLpro	Docking	−7.2	44
Isochaetochromin D1	3CLpro	Docking	−7.9	44
Aspergilol H (R)	3CLpro	Docking	−7.9	44
Aspergilol H (S)	3CLpro	Docking	−8.3	44
11a-Dehydroxyiso-terreulactone A	3CLpro	Docking	−8.9	44
Arisugacin A	3CLpro	Docking	−8.0	44
Aspernolide A	3CLpro	Docking	−7.1	44
Rhodatin	3CLpro	Docking	−7.1	44
Scedapin C	3CLpro	Docking	−8.6	44
Scequinadoline A	3CLpro	Docking	−8.7	44
14S-Oxoglyantrypine	3CLpro	Docking	−8.4	44
Deoxynortryptoquivalin e	3CLpro	Docking	−7.6	44
Quinadoline B	3CLpro	Docking	−8.3	44
Norquinadoline A	3CLpro	Docking	−8.1	44

Asperterrestide A (S)	3CLpro	Docking	−8.3	44
Asperterrestide A (R)	3CLpro	Docking	−8.4	44
Rubrolide S	3CLpro	Docking	−7.7	44
Isoaspulvinone	3CLpro	Docking	−7.4	44
Pulvic acid	3CLpro	Docking	−6.6	45
Demethyloleuropein	3CLpro	Docking	-8.9	45
Neo–nüzhenide	3CLpro	Docking	-8.7	45
Nüzhenide	3CLpro	Docking	-8.6	45
Lopinavir	3CLpro	Docking	-7.8	45

STR* = S-protein trimerization region; S-RBD* = S-Protein Receptor Binding Domain; p.w.* = present work

Table S5. Quantitative comparison between our compounds (highlighted in bold) and other ligands proposed as SARS-CoV-2 like-inhibitor targeting viral S-Protein and 3CLpro.

References

1. Spadoni, G.; Bedini, A.; Bartolucci, S.; Pala, D.; Mor, M.; Riccioni, T.; Borsini, F.; Cabri, W.; Celona, D.; Marzi, M.; et al. Towards the development of 5-HT7 ligands combining serotonin-like and arylpiperazine moieties. *Eur. J. Med. Chem.* **2014**, doi:10.1016/j.ejmech.2014.04.034.

2. Singh, S.; Chauhan, P.; Ravi, M.; Yadav, P.P. Eosin Y-Yb(OTf)3 catalyzed visible light mediated electrocyclization/indole ring opening towards the synthesis of heterobiaryl-pyrazolo[3,4-: B] pyridines. *New J. Chem.* **2018**, doi:10.1039/c8nj00862k.
